# Strain-time curve analysis by speckle tracking echocardiography in cardiac resynchronization therapy: Insight into the pathophysiology of responders vs. non-responders

**DOI:** 10.1186/s12947-016-0057-4

**Published:** 2016-04-18

**Authors:** Andrew C. Y. To, Rodolfo D. Benatti, Kimi Sato, Richard A. Grimm, James D. Thomas, Bruce L. Wilkoff, Deborah Agler, Zoran B. Popović

**Affiliations:** 1 Section of Cardiovascular Imaging, Department of Cardiovascular Medicine, Cleveland Clinic, 9500 Euclid Avenue, Cleveland, OH USA; 2 Section of Electrophysiology, Department of Cardiovascular Medicine, Cleveland Clinic, 9500 Euclid Avenue, Cleveland, OH USA; 3 Department of Cardiology, North Shore Hospital, 124 Shakespeare Rd, Takapuna, Auckland New Zealand

**Keywords:** Cardiac resynchronization therapy, Left bundle branch block, Dyssynchrony

## Abstract

**Background:**

Patients with non-ischemic heart failure etiology and left bundle branch block (LBBB) show better response to cardiac resynchronization therapy (CRT). While these patients have the most pronounced left ventricular (LV) dyssynchrony, LV dyssynchrony assessment often fails to predict outcome. We hypothesized that patients with favorable outcome from CRT can be identified by a characteristic strain distribution pattern.

**Methods:**

From 313 patients who underwent CRT between 2003 and 2006, we identified 10 patients who were CRT non-responders (no LV end-systolic volume [LVESV] reduction) with non-ischemic cardiomyopathy and LBBB and compared with randomly selected CRT responders (*n* = 10; LVESV reduction ≥15 %). Longitudinal strain (ε_long_) data were obtained by speckle tracking echocardiography before and after (9 ± 5 months) CRT implantation and standardized segmental ε_long_-time curves were obtained by averaging individual patients.

**Results:**

In responders, ejection fraction (EF) increased from 25 ± 9 to 40 ± 11 % (*p* = 0.002), while in non-responders, EF was unchanged (20 ± 8 to 21 ± 5 %, *p* = 0.57). Global ε_long_ was significantly lower in non-responders at pre CRT (*p* = 0.02) and only improved in responders (*p* = 0.04) after CRT. Pre CRT septal ε_long_ -time curves in both groups showed early septal contraction with mid-systolic decrease, while lateral ε_long_ showed early stretch followed by vigorous mid to late contraction. Restoration of contraction synchrony was observed in both groups, though non-responder remained low amplitude of ε_long_.

**Conclusions:**

CRT non-responders with LBBB and non-ischemic etiology showed a similar improvement of ε_long_ pattern with responders after CRT implantation, while amplitude of ε_long_ remained unchanged. Lower ε_long_ in the non-responders may account for their poor response to CRT.

## Background

Cardiac resynchronization therapy (CRT) improves survival by stopping or reversing adverse remodeling that occurs in systolic heart failure [1–3]. The mechanism of CRT is coordination of the contraction pattern of otherwise dyssynchronous opposing left ventricular (LV) walls which are, in a clinically common setting of left bundle branch block (LBBB), septal and lateral ones [4, 5].

While several clinical parameters have been well established as predictors of CRT response [1, 3, 6], failure of CRT occurs even if all of clinical parameters predict otherwise. It is unclear if this is due to a specific contraction pattern of these patients, or some other factors. For this purpose we analyzed the contraction pattern of CRT patients with clinical and procedural characteristics known to be associated with pronounced reverse remodeling response: non-ischemic heart failure etiology, LBBB pattern on electrocardiogram (ECG), QRS duration on ECG >140 ms, and LV electrode located over the mid/base lateral or posterolateral wall [1, 3, 6, 7]. We identified a group of patients who, despite these favorable pre-procedural characteristics, did not show reverse remodeling, and compared it to a group with good CRT response using segmental strain analysis.

## Methods

### Population

**Fig 1:**
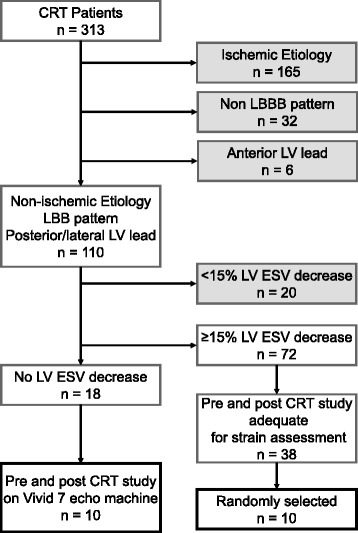
Flow diagram of patient selection process. CRT, cardiac resynchronization therapy; LBBB, left bundle branch block; LV, left ventricular; LVESV, left ventricular end-systolic volume

The study population was selected from 313 consecutive heart failure patients who underwent implantation of a biventricular device at the Cleveland Clinic between January 2003 and June 2006, who also had long-term echocardiographic follow-up [3] (Fig. 1). They all had symptomatic heart failure, an ejection fraction of <35 % and QRS duration of >120 ms. CRT was provided in the standard fashion with 3 trans-venous leads, including the LV lead inserted through the coronary sinus.

From those who had a complete baseline echocardiographic study performed within 3 months before device implantation on a Vivid 7 system (GE Healthcare, Horten, Norway) and with echocardiographic follow-up (>3 months), we identified 10 patients who were CRT non-responders (unchanged or increased LV end-systolic volume [LVESV] at follow-up) with the following clinical characteristics: non-ischemic cardiomyopathy, LBBB, and LV lead placed over the base/mid posterior or lateral LV wall. We compared this group with a group of ten patients with the same characteristics except for being CRT responders (reduction of LVESV at follow-up ≥15 %) who were randomly selected from the same study population. The patient data were de-identified and that the study was approved by the Cleveland Clinic Institutional Review Board.

### Longitudinal strain-time analysis

Two-dimensional speckle tracking (EchoPAC 10.0, GE Healthcare) was performed using the images acquired in the apical 4 chamber, 2 chamber and long axis views. The peak of the R wave on the ECG was used as a reference time point for end-diastole, and aortic valve closure was used as a time point for end-systole. Segmental longitudinal strain (ε_long_) curves derived from a single cardiac cycle were exported for analysis, with resulting 18 ε_long_ curves corresponding to the basal-mid-apical septum, anteroseptum, inferior, posterior, lateral and anterior segments obtained in each patient. To correct for RR interval variation, ε_long_ curves were normalized using the two reference time points of end-diastole and end-systole, so that time was expressed as a percentage of systole (% systole) [8, 9]. Care was taken to ensure that the end-diastolic time points for speckle tracking are consistent across all three apical views.

**Fig 2:**
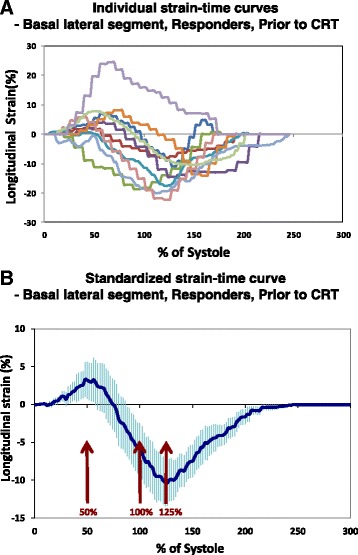
Individual (**a**) and averaged (**b**) strain-time curve of basal lateral segment in the responders prior to cardiac resynchronization therapy. **a** Individual segmental strain-time curves, normalized for systolic duration, obtained from basal lateral segment in the responders group prior to start of cardiac resynchronization therapy. Systolic duration is defined as the time from the mitral valve closure to aortic valve closure. **b** Averaged normalized segmental strain-time curve (with error bars indicating standard error) obtained by averaging the data shown in Panel **a**. Arrows depict the time points corresponding to mid systole (50 %), end-systole (100 %) and post-systole (125 %). CRT, cardiac resynchronization therapy

To better characterize segmental ε_long_ profiles in both patient groups pre and post CRT, individual ε_long_ curves were averaged to obtain a characteristic average ε_long_ profile [8, 9]. Figure 2 shows individual ε_long_ curves of the basal anterolateral segment (A), and corresponding group average ε_long_ curve (B) obtained in CRT responders before pacing. Finally, global ε_long_ was calculated by averaging segmental values.

### Statistical analysis

Continuous variables with normal distribution were expressed as mean ± SD, and categorical variables were expressed as count and percentage. Between and within-group differences were assessed by Wilcoxon signed-rank test and Mann-Whitney *U* test as appropriate. To assess opposing wall mechanics before and after CRT, we entered ε_long_ measurements from basal and mid LV levels of the opposing walls (e.g., septal and lateral) obtained at early systole (50 % of systole duration), end-systole (100 % of systole duration) and post-systole (125 % of systole duration) into mixed model analysis, using an unstructured covariance model. The model was constructed with “time” (% systole) as covariate, and “wall” (e.g., septum vs. lateral) and “group” (responders vs. non-responders) as factors. Interaction between time and wall, which quantitates between-wall difference in pattern of ε_long_ development, was taken to represent loss of coordinated contraction (dyssynchrony). The Akaike criterion was used to determine the optimal model. P values <0.05 were considered as statistically significant. Statistical analyses were performed using JMP Pro 10.0.2 (SAS Institute Inc., Cary, North Carolina).

## Results

Of the 313 consecutive patients who underwent CRT and follow-up, 155 (50 %) had reverse remodeling and 89 (28 %) patients had no response to CRT. Of those 89 patients, 10 (11 %) patients met the inclusion criteria. Among patients who responded to CRT, 38 patients met the inclusion criteria and 10 patients were randomly selected as the comparison group. The baseline clinical, electrocardiographic, and echocardiographic characteristics were similar between responders and non-responders (Table 1). One non-responder patient was excluded from the analysis because of poor images at baseline. The mean age of the population was 58 ± 8 years, with 89 % having New York Heart Association (NYHA) class III/IV symptoms. All patients had sinus rhythm and left bundle branch block with a QRS duration of 168 ± 20 ms. All implanted devices were biventricular implantable cardioverter defibrillators (CRT-D), with left ventricular lead position in the lateral (*n* = 12) and posterolateral (*n* = 7) cardiac veins. There was no difference between these two groups.

**Table 1:** Clinical data in two patient groups

	Responders	Non-responders	*P* value
n	10	9	
Age (years)	57 ± 11	60 ± 6	0.496
Female Gender, n (%)	5 (50 %)	4 (44 %)	0.809
Weight (kg)	87 ± 18	91 ± 19	0.669
BSA (m^2^)	2.0 ± 0.3	2.1 ± 0.3	0.632
LBBB, n (%)	10 (100 %)	9 (100 %)	NA
Non-ischemic etiology, n (%)	10 (100 %)	9 (100 %)	NA
Sinus rhythm, n (%)	10 (100 %)	9 (100 %)	NA
CRT-D, n (%)	10 (100 %)	9 (100 %)	NA
Follow up from implant (months)	8.5 ± 4.8	9.0 ± 5.2	0.832
NYHA III/IV – pre, n (%)	8 (80 %)	9 (100 %)	0.776
NYHA III/IV – post, n (%)	2 (20 %)	5 (55 %)	0.109
QRS width (ms) – pre	167 ± 18	169 ± 22	0.789
QRS width (ms) – post	153 ± 18	157 ± 26	0.771

Values are mean ± SD or n (%). *BSA* body surface area, *LBBB* left bundle branch block, *CRT*-*D* cardiac resynchronization therapy defibrillator, *NYHA* New York Heart Association

Baseline left ventricular volumes and ejection fraction were not different between responders and non-responders (Table 2). At follow-up after an average CRT duration of 9 ± 5 months, responders showed improvement of LV end-diastolic volume (LVEDV) (*p* = 0.01), LVESV (*p* = 0.002), and EF (*p* = 0.002), while non-responders showed significant enlargement of LVEDV (*p* = 0.004) and LVESV (*p* = 0.01). Although there was no difference in LV volumes or ejection fractions between the two groups before CRT, LVEDV (*p* = 0.008) and LVESV (*p* = 0.002) were significantly smaller, and EF (*p* = 0.002) was higher in responders after CRT implantation.

**Table 2:** Left ventricular volumes and ejection fraction pre- and post-cardiac resynchronization therapy

	Responders	Non-responders	*p*-value
Pre			
LVEDV (ml)	241 ± 81	310 ± 111	0.11
LVESV (ml)	184 ± 74	248 ± 109	0.13
LVEF (%)	25 ± 9	21 ± 8	0.37
Post			
LVEDV (ml)	180 ± 85**	341 ± 118**	0.008
LVESV (ml)	111 ± 63**	274 ± 113*	0.002
LVEF (%)	40 ± 11**	21 ± 5	0.002

*: *p* < 0.05 versus pre; **: *p* < 0.01 versus pre

*LVEDV* left ventricular end-diastolic volume, *LVESV* left ventricular end-systolic volume, *LVEF* left ventricular ejection fraction

### Global ε_long_

Table 3 outlines the global ε_long_ values in responders and non-responders at mid-systole (50 % systole), end-systole (100 % systole) and early post-systole (125 % systole). Before CRT, global ε_long_ at end-systole (*p* = 0.02) and early post-systole (*p* = 0.03) were lower in the non-responder group. At a follow up, in addition to end-systole and early post-systole ε_long_, non-responders showed lower global mid-systole ε_long_ (*p* = 0.03)_._ In responders, global end-systolic (*p* = 0.04) and early post-systolic ε_long_ (*p* = 0.03) demonstrated improvement after CRT implantation, whereas there were no significant improvement in non-responders.

**Table 3:** Comparison of longitudinal global strain (ε_long_) in responders and non-responders at mid-systole (50 % systole), end-systole (100 % systole) and early post-systole (125 % systole)

	Responders			Non-responders		
% systole	50 %	100 %	125 %	50 %	100 %	125 %
Pre	-1.68 ± 0.78	-5.97 ± 2.73	-6.11 ± 3.05	-0.47± 0.46	-2.91 ± 0.96†	-3.48 ± 1.58†
Post	-2.49 ± 0.9	-9.94 ± 3.11*	-9.27 ± 4.08*	-0.17 ± 0.58†	-3.69 ± 2.47‡	-4.39 ± 3.22†

*: *p* < 0.05 versus pre; †: *p* < 0.05 versus Responders; ‡: *p* < 0.001 versus Responders

ε_long,_ longitudinal global strain

### Opposing walls mechanics before and after CRT

**Fig 3:**
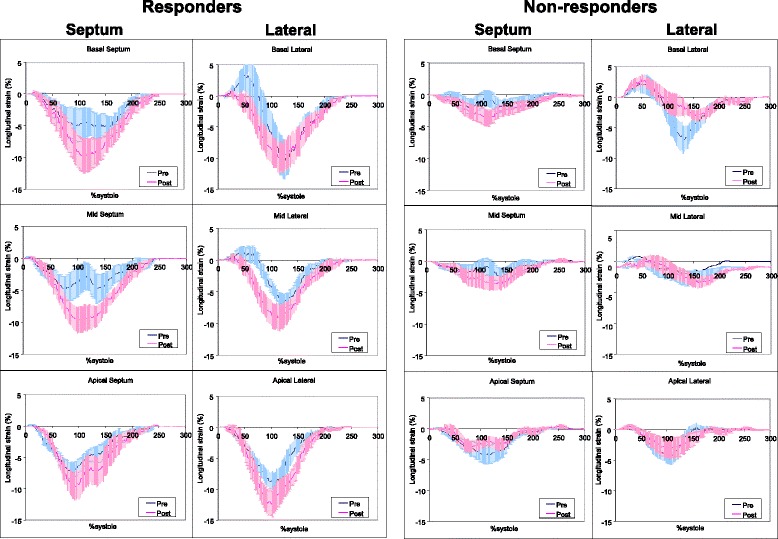
Averaged normalized segmental longitudinal strain-time curves of septal and lateral segments in responders and non-responders to cardiac resynchronization therapy. The strain-time curves have lower amplitude in non-responders. Cardiac resynchronization therapy results in a more uniform shape of the strain-time curves with cardiac resynchronization therapy. Error bars represent standard errors. Pre: prior to start of cardiac resynchronization therapy; Post: 9 ± 5 months after the start of cardiac resynchronization therapy

**Fig 4:**
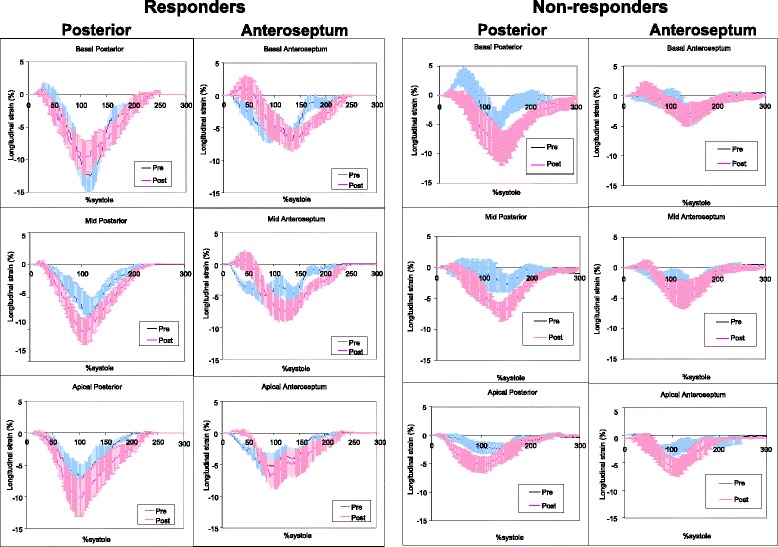
Averaged normalized segmental longitudinal strain-time curves of posterior and anteroseptal segments in responders and non-responders to cardiac resynchronization therapy. Error bars represent standard errors. Pre: prior to start of cardiac resynchronization therapy; Post: 9 ± 5 months after the start of cardiac resynchronization therapy

**Fig 5:**
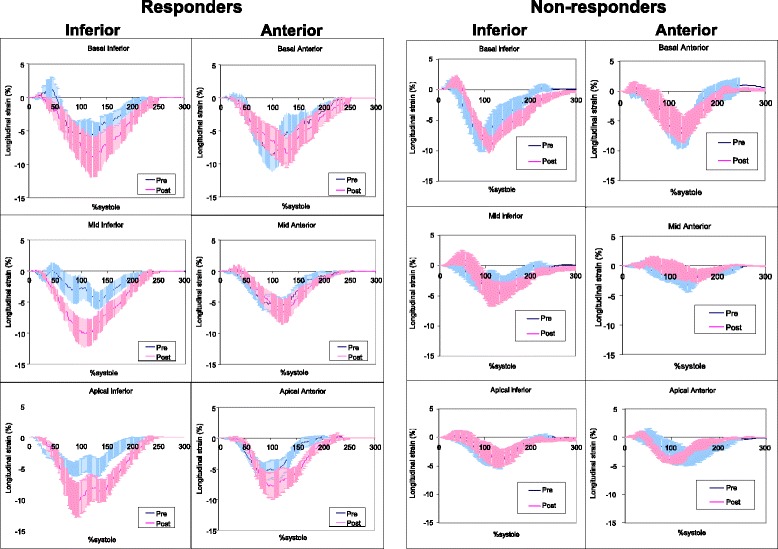
Averaged normalized segmental longitudinal strain-time curves of inferior and anterior segments in responders and non-responders to cardiac resynchronization therapy. Error bars represent standard errors. Pre: prior to start of cardiac resynchronization therapy; Post: 9 ± 5 months after the start of cardiac resynchronization therapy

Figure 3 showed averaged ε_long_-time curves of 18 segments in responder and non-responder. Each panel showed pre- and post-CRT septal and lateral wall ε_long_-time curves of basal, mid, and apical segment for responders and non-responders. Pre- CRT, the basal and mid septal ε_long_-time curves demonstrated a pattern of early septal contraction with mid-systolic decrease, while basal and mid lateral ε_long_-time curves demonstrated an early stretch followed by vigorous mid to late contraction. Post-CRT, restoration of contraction synchrony was noted in both the septal and lateral segments. This pattern pre- and post-CRT was to a lesser extent observed in the posterior and anteroseptal walls respectively (Fig. 4) and was lost in the inferior and anterior walls (Fig. 5). While the shapes of the ε_long_-time curves were similar in responders and non-responders, these two groups differed markedly in amplitude, both pre- and post-CRT.

To quantify these observations, we compared ε_long_ of the opposing walls at mid, end, and post-systole (i.e., 50 %, 100 % and 125 % of systole).

#### Septal vs. lateral

**Fig 6:**
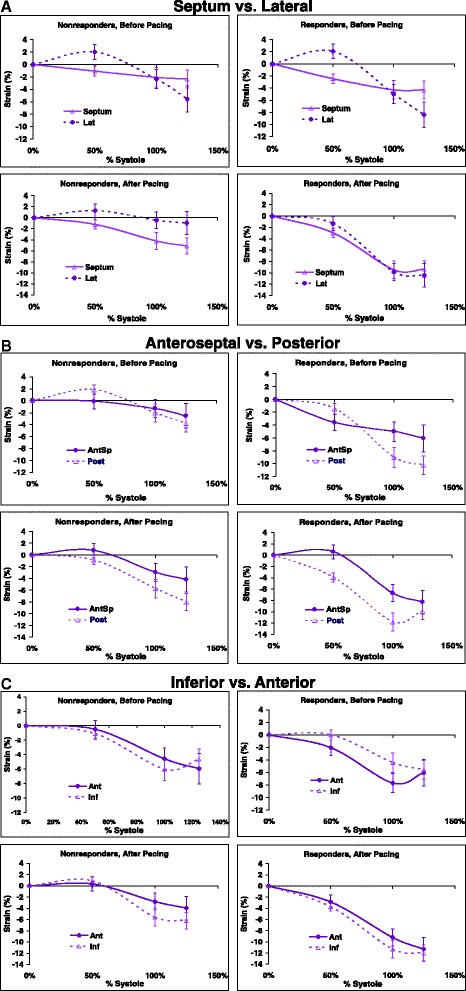
Comparison of opposing wall mechanics during ventricular contraction before and after cardiac resynchronization therapy (CRT). Strain values are measured at mid-systole (50 %), end-systole (100 %) and post-systole (125 %) before (upper panels) and >3 months after the start of CRT (lower panels). **a** Septal and lateral longitudinal wall strains; **b** Anteroseptal and posterior longitudinal wall strains; and **c** Inferior and anterior longitudinal wall strains. Error bars represent standard errors. CRT, cardiac resynchronization therapy

Prior to CRT, ε_long_ was lower in the lateral than in the septal wall (*p* = 0.001). Septal and lateral walls were also different in the pattern of ε_long_ increase (*p* = 0.001; Fig. 6a). There was no difference between responders and non-responders in overall ε_long,_ the pattern of ε_long_ increase, or in the difference between ε_long_ of opposing walls (*p* = NS for all). After CRT, lateral wall ε_long_ was still higher (*p* = 0.007), but difference in the pattern of ε_long_ increase disappeared (*p* = 0.80). Responders showed higher overall ε_long_ (*p* = 0.02), and more marked ε_long_ increase over time (*p* = 0.03).

#### Posterior vs. anteroseptal

Posterior and anteroseptal walls prior to CRT showed different patterns of ε_long_ increase (*p* = 0.01), but similar average ε_long_ (*p* = 0.20) (Fig. 6b). Interestingly, responders had higher overall ε_long_ of these two walls (*p* = 0.02). After 12 months of CRT, difference in the pattern of ε_long_ increase in opposing walls disappeared (*p* = 0.70). Responders showed a trend towards having higher overall ε_long_ (*p* = 0.08).

#### Inferior vs. anterior

Inferior and anterior walls before CRT had similar patterns of ε_long_ increase (*p* = NS) (Fig. 6c). Responders and non-responders did not differ in the overall ε_long_, the pattern of ε_long_ increase, or in the difference between opposing wall ε_long_ (*p* = NS for all). After 12 months of CRT, responders showed higher overall ε_long_ (*p* = 0.02), again with no difference between ε_long_ of opposing walls (*p* = NS).

### Predicting CRT response with baseline Global ε_long_

In the logistic regression model, the pre CRT average ε_long_ was significantly associated with CRT response. The area under the receiver operating characteristic curve showed average ε_long_ at end systole -4.0 % as optimal cutoff point (AUC 0.83, 95 % CI 0.64 – 1.00, *p* = 0.014). This cutoff point predicted CRT response with 78 % specificity and 80 % sensitivity in this population. Other echocardiographic parameters and baseline characteristics did not predict response.

## Discussion

In this paper, CRT non-responders despite having favorable predictors of good response to CRT (i.e., non-ischemic heart failure etiology, LBBB, QRS complex duration >140 ms, and appropriate LV electrode placement) have lower global end systolic longitudinal strain but a similar pattern of longitudinal strain contraction heterogeneity pre-CRT as responders. This indicates that the presence of, and subsequent improvement of, dyssynchrony is not sufficient to result in reverse remodeling during CRT. Their markedly lower longitudinal strain suggested myocardial dysfunction burden might be the predictor of reverse remodeling in patients with non-ischemic cardiomyopathy.

### Ventricular function in left bundle branch block: characteristic contraction pattern versus dyssynchrony

Animal models have demonstrated that right ventricular pacing (an LBBB surrogate) induces a characteristic pattern of blunted early septal and forceful delayed lateral wall contraction preceded by its early stretch [4, 5, 10]. Traditional dyssynchrony indices are positive scalar numbers that lack the ability to localize the origin of dyssynchrony. As an example, the standard deviation of the time to peak of systolic myocardial tissue velocities [11] will have the same value whether the most delayed segment is in the basal lateral or apical septal LV segment. Some clinical studies have shown that early segmental stretch can be detected in some CRT candidates [12, 13]. In this study, we constructed average segmental strain-time curves from two well-defined patient groups, a characteristic pattern of early, suppressed contraction of the basal and mid septum, and early stretch followed by strong contraction of the basal and mid lateral walls. These characteristic segmental strain-time curves overcome the major drawback of LV dyssynchrony indices [13–18] in defining the characteristic profile of LBBB induced dyssynchrony.

The improvement of LV contraction heterogeneity underlies some of the benefits of CRT. Unfortunately, prospective multi-institutional trials failed to confirm the predictive value of the most frequently used dyssynchrony indices [10, 19]. Even in selected populations that qualify for CRT therapy by current criteria, the predictive value of a dyssynchrony index may be low [19, 20]. Several other factors make relevance of dyssynchrony indices doubtful. Dyssynchrony detected by one index is often not confirmed when a different index is used [21]. Dyssynchrony indices are often positive in the setting of narrow QRS complex [21], where CRT treatment is shown to be ineffective [22, 23]. Velocity-based indices are influenced by myocardial translational motion that is often present in the setting of severe LV dilatation [24]. Finally, the limit of any dyssynchrony index may in the end be the fact that in some patients, myocardium has simply “burnt-out,” hence losing therapeutic and contractile reserve [25]. Similar characteristics of strain-time and their improvement were observed both in responder and non-responder groups in this study and did not predict LV reverse remodeling after CRT implantation. However baseline average longitudinal strain might be a surrogate to distinguish the non-responder group.

### LV longitudinal strain as predictors of reverse remodeling

The MADIT-CRT cohort also supported that decreased average longitudinal strain predicts less beneficial effects of CRT, especially in the setting of LBBB [26, 27]. Several studies reported baseline global longitudinal strain predicts LV reverse remodeling after CRT in patients with both ischemic and non-ischemic cardiomyopathy [26, 28]. Our result confirmed these reports by demonstrating it in CRT non-responders with the non-ischemic population, though a small sample size. A recent study showed depressed longitudinal strain was strongly associated with total scar burden assessed by cardiovascular magnetic resonance imaging in ischemic heart failure patients, and it may be a sensitive parameter of LV contractile reserve and the presence of viable myocardium [28–30]. Another report showed longitudinal strain improvement after CRT implantation also indicated better clinical outcome and reverse remodeling, suggesting contractile reserve is associated with reverse remodeling [31]. Since responders showed significant improvement of longitudinal strain in the present study, our findings suggest that underlying myocardial deformation could be the determinant of reverse remodeling after CRT implantation in patients with non-ischemic cardiomyopathy and LBBB.

### Study limitations

This is a small, retrospective, observational study. Only longitudinal strains were assessed, although various previous studies have used circumferential or radial strains [5, 16]. However, longitudinal strains have lower measurement error, and the ability of obtaining anatomically accurate views is often easier from the apical rather than from the parasternal position. In addition, as we used very strict selection criteria, the number of patients was relatively small, and we lacked statistical power to perform multivariate analysis to predict CRT response. Therefore response to CRT might be affected by other factors besides LV strain. Additionally, our result seems inefficient in patients with ischemic cardiomyopathy, who often do not show reverse remodeling after CRT. Further large prospective study is required to verify the predictive value of longitudinal strain in assessing LV reverse remodeling.

## Conclusions

Our study defines the characteristic segmental pattern of LV contraction in patients with non-ischemic cardiomyopathy and LBBB before and after CRT. CRT non-responders with non-ischemic cardiomyopathy and LBBB demonstrated a qualitatively similar segmental contraction pattern but have dramatically decreased longitudinal strain. These findings may help in predicting the outcome of CRT in these patients.

## Abbreviations

CRT: cardiac resynchronization therapy
LV: left ventricular
LBBB: left bundle branch block
LVESV: left ventricular end-systolic volume
EF: ejection fraction
ε_long_: Longitudinal strain
ECG: electrocardiogram
NYHA: New York Heart Association
CRT-D: biventricular implantable cardioverter defibrillator
LVEDV: left ventricular end-diastolic volume
